# Notes and News

**Published:** 1895-11-16

**Authors:** 


### Notes and News.

(Collected and Communicated.)

A dinner was given in honour of Sir Thornley Stoker, President of the Royal College of Surgeons in Ireland, last week, to celebrate the occasion of his receiving the honour of knighthood.

The Bishop of Winchester opened a cottage hospital at Farnham last week. The hospital has been built from the proceeds of a legacy by the late Mr. George Trimmer. The building is of red brick, with stone facings. It contains two wards, accident and operation room. It is well situated, facing the road, with a southern aspect.

The report of Dr. Frazer and Dr. Lauder Brunton on the condition of Dr. Cornelius Herz leaves no doubt as to the serious nature of his malady. Both doctors, and others of eminence and authority, have pronounced that he would be unable to travel without great danger to his life. His heart symptoms are very distressing, and he is unable to sit up without a sensation of faintness.

We are glad to learn that the Poplar Hospital for Accidents has benefited to the extent of £300 through Sir W. Pearson's kindness in permitting the Blackwall Tunnel to be opened to the public on its behalf last week, as we mentioned in our last issue. Part of the money collected was represented by 40,000 coppers, and some difficulty arose in transferring them to the hospital. Here, as in other emergencies, the ambulance was ready to help, and the coins were carefully placed upon it, the hood drawn over all, and the precious burden thus conveyed to the hospital. The conductors amused themselves by replying to the curious crowd that they conveyed a dead "copper," which caused no small following to attend. We learn that the special benefit arranged by the working people of Canning Town is likely to result in £100 being handed over to the hospital.

The Societies of Agriculture and of Veterinary Medicine of Milan have opened subscription lists for the erection of a statue to Pasteur. Scientific men in America are forming a committee to co-operate with Great Britain in establishing a memorial of Huxley.

Baron Larrey, the great French military surgeon who died recently, came of a family identified with the Napoleonic dynasty for nearly a hundred years. Baron Larrey's father was the renowned surgeon of the armies of Napoleon the First.

The opening by the Duke of Devonshire of the new home (the "Passmore Edwards House") at the colony of the National Society for the Employment of Epileptics, at Chalfont St. Peter, which was postponed from August 7th last owing to his Grace being summoned to Osborne on that day, has now been fixed to take place on the 26th inst., at a quarter to three p.m.

The Carrington Convalescent Hospital, at Camden, New South Wales, has an admirable arrangement for the comfort of patients. This is a specially-constructed railway carriage for the conveyance of patients. It runs from Sydney twice a week, a hospital nurse being in charge. The hospital, which was the first of its kind in New South Wales, is lamenting the death of its generous founder, Mr. Paling. The institution, which is built of brick, stands on rising ground, and will accommodate 94 patients.

The Laundry and Sanitary Exhibition opened last week at the Agricultural Hall, Islington, is the third successfully organised by Messrs. Cordingley, the proprietors of *Laundry News*, and has proved of even greater interest to those concerned in such matters than its predecessors. The development of steam laundries has been little short of wonderful of late years, and still the number of new inventions and improvements grows ever greater. We hope to give next week a more detailed account of some of the very interesting exhibits.

Mr. Robert H. Bax, one of the governors of the Great Northern Central Hospital, has sent us a circular in which he states: "Huge sums have been paid in commission to the late secretary, Mr. W. T. Grant, over and above his salary, and such commission has been most improperly dealt with in reports, thereby deceiving the governors. Thus, in the report of 1894, page 19, of the item headed Sundries, Alterations, and Repairs, £592 9s. 11d., the late secretary received £519 6s. 9d., being 2½ per cent. on £20,774, of which legacies amounted to £16,902." Mr. Bax states that the total remuneration paid to the secretary was £1,011 4s. 9d. for a single year. The committee of management have refused to convene a special meeting of the Governors, to whom Mr. Bax (67, Finsbury Park Road, N.) now appeals for co-operation. The chairman of the Committee of Management must be held primarily responsible for these extraordinary payments, and he ought to be called upon for an explanation at a special meeting of the Governors, which should be held without a moment's delay. Any governor agreeing with these views should send his name to Mr. Bax, to be added to the requisition summoning a special meeting of the Governors under the rules.

